# Preliminary Purification and Partial Characterization of a Functional Bacteriocin of *Lacticaseibacillus paracasei* Zhang and Mining for its Gene Cluster

**DOI:** 10.1007/s12602-024-10249-9

**Published:** 2024-05-15

**Authors:** Tian Huang, Zhaojie Li, Xinan Qu, Guoqiang Yao, Lai-Yu Kwok, Qiuwen He, Heping Zhang

**Affiliations:** 1 Key Laboratory of Dairy Biotechnology and Engineering, Ministry of Education, Inner Mongolia Agricultural University, Hohhot, 010018 China; 2 Key Laboratory of Dairy Products Processing, Ministry of Agriculture and Rural Affairs, Inner Mongolia Agricultural University, Hohhot, 010018 China; 3 Inner Mongolia Key Laboratory of Dairy Biotechnology and Engineering, Inner Mongolia Agricultural University, Hohhot, 010018 China; 4 College of Food Science and Engineering, Qingdao Agricultural University, Qingdao, 266109 Shandong China; 5 Qingdao Special Food Research Institute, QingdaoShandong, 266109 China; 6 Department of College of Food Science and Engineering, Inner Mongolia Agricultural University, No. 306, Zhaowuda Road, Saihan District, Hohhot, 010018 Inner Mongolia China

**Keywords:** *Lacticaseibacillus paracasei* Zhang, Antibacterial metabolite, Purification, Characterization, Bacteriocin

## Abstract

Bacteriocins produced by lactic acid bacteria (LAB) have good potential for use as food biopreservatives. *Lacticaseibacillus paracasei* Zhang (*L. paracasei* Zhang) is both a food use and a probiotic bacterium. This study aimed to purify and preliminary characterize the active antibacterial metabolite of *L. paracasei* Zhang. The cell-free supernatant of *L. paracasei* Zhang was collected and purified by ultrafiltration and gel filtration chromatography. The 1–3 kDa active fraction could inhibit the growth of *Staphylococcus aureus* but not *Escherichia coli*. Further antibacterial activity assays revealed its capacity to suppress various foodborne and human opportunistic pathogens (including *Staphylococcus aureus*, *Pseudomonas fluorescens*, *Pseudomonas aeruginosa*, *Listeria monocytogenes*, and *Bacillus cereus*), but not fungi. The antibacterial activity showed good tolerance to heat (40 to 100 °C), acid–base (pH 2–3 and pH 6–10), and digestions by a number of industrial and animal/human enzymes (such as trypsin, pepsin, α-amylase, and protease K, except papain); these desired properties make it a suitable biopreservative to be used in harsh and complex industrial production processes. The high papain sensitivity suggested a proteinaceous/peptide nature of the bioactivity. Moreover, our genomic data mining for bacteriocin through BAGEL4 revealed an area of interest encoding a complete set of putative genes required for bacteriocin production. In conclusion, our study showed that *L. paracasei* Zhang can produce extracellular functional antibacterial metabolite, likely a class II bacteriocin. Our preliminary extraction and characterization of the active metabolite demonstrated that it has good potential to be used as a biopreservative or an agent for suppressing gastrointestinal infections.

## Introduction

Food preservatives are often added to food products to prevent food spoilage [1]. However, with the improvement of living standards and consumers’ health awareness, chemical preservatives are increasingly rejected by consumers because of their potential toxicity or negative health effects [2]. Therefore, the development of natural LAB biological preservatives with a broad spectrum of antimicrobial activity, stability, and safety has attracted much attention from the food industry [3]. Biopreservatives have the potential for use in the natural control of food spoilage microorganisms and foodborne pathogens. Thus, it is of interest to isolate microbes that can synthesize these active biomolecules, as well as to extract and characterize the produced antibacterial metabolites [4].

LAB are ubiquitous lactic acid–producing gram-positive bacteria. In the process of growth and metabolism, LAB can secrete a variety of metabolites, such as organic acids, diacetyl, ethanol, hydrogen peroxide, exopolysaccharides, and bacteriocins, many of which show good antimicrobial effects [5, 6]. For example, it was found that L-phenyl lactic acid extracted from the fermentation supernatant of *Lactiplantibacillus plantarum* Z316 could inhibit the food pathogen *Salmonella enterica* subsp. *enterica* ATCC 14028 [7]. Another study reported the inhibitory activity of some polysaccharide components in the supernatant of *Pseudoalteromonas haloplanktis* TAC125 against *Staphylococcus epidermidis* biofilm formation [8]. The bacteriocin M1-UVs300 isolated from *Lactiplantibacillus plantarum plantarum* M1-UVs300 exhibited a broad-spectrum antibacterial activity against both Gram-positive and Gram-negative bacteria [9].

Among the antimicrobial materials, bacteriocins have attracted much attention because of their enticing features, such as good safety, high efficacy in microbe suppression, non-toxicity, residue-free, low/no risk in causing drug resistance, and ease of decomposition by protease after being ingested. Bacteriocins are antibacterial peptides or precursors synthesized by ribosomes in the process of bacterial growth and metabolism for enhancing survival competitiveness by suppressing surrounding microorganisms [10], and some of which have shown strong inhibitory effects against antibiotic-resistant bacteria [11]. It was reported that most bacteria and archaea produce at least one bacteriocin [12]. Unlike synthetic drugs and chemical preservatives, bacteriocins have a rapid and powerful effect even at low doses, and they do not tend to drastically alter the natural human gut commensals [3, 13]. Furthermore, most bacteriocins have good heat, acid, and alkali resistances but are protease-sensitive [14]. Their modes of action, high stability, and desired inhibitory effects on potential pathogens and drug-resistant bacteria have made them a good option for use in food spoilage control and fresh-keeping [15].

The *Lacticaseibacillus paracasei* (*L. paracasei*) species has been widely used in the food industry, and some members of this species are known to produce antibacterial metabolites and/or bacteriocins such as paractocin SD1 and bacteriocins BGSJ2-8 and paracin 54 [16–18]. Some *L. paracasei*-originated bacteriocins have been shown to exert intestinal homeostasis and regulatory effects [19].

*L. paracasei* Zhang strain was isolated from a naturally fermented horse milk sample collected in Xilingol grassland, Inner Mongolia, in 2001 [20]. This strain has been shown to have good fermentation characteristics and various probiotic effects through in vitro, animal, and clinical intervention trial studies. For example, it could increase antioxidation and anti-lipid peroxidation activities [21], enhance cellular/humoral immunity and tumor suppressive function [22], improve blood lipids and liver lipid metabolism [23], prevent type II diabetes, and raise liver function and protect against liver injury [24]. Moreover, in population trials, it has shown effective gut microbiota regulatory effects by enhancing the beneficial bacteria while suppressing the potentially harmful ones [21] and prophylactic effects against upper respiratory tract infection [25].

This study found that the genome of *L. paracasei* Zhang had distinct bacteriocin gene cluster-encoding genomic regions. Thus, the antibacterial activities of *L. paracasei* Zhang were explored by extracting, preliminarily purifying, and characterizing its extracellular antibacterial metabolite. The antimicrobial spectrum of the bioactive extract against indicator bacteria and fungi was also determined. Our results showed that *L. paracasei* Zhang produces a functional and stable bacteriocin with a broad antimicrobial activity spectrum, which is a novel microbial resource of potential for use as a food biopreservative.

## Materials and Methods

### Microbial Strains and Cultivation

The *L. paracasei* Zhang was isolated from the traditional fermented horse milk (pH 3.37–3.94) in Zhenglan Banner, Xilingol, Inner Mongolia in 2001 [20]. It is preserved at the Key Laboratory of Dairy Biotechnology and Engineering of the Ministry of Education, Inner Mongolia Agricultural University. The frozen bacterial stock was activated and inoculated in liquid de Man, Rogosa, and Sharpe (MRS) medium (pH 5.90) and aerobically cultured at 37 °C for 24 h. The indicator strains of the antimicrobial assay are shown in Table 1. *Actinobacillus actinomycetemcomitans* (*A. actinomycetemcomitans*), *Fusobacterium nucleatum* subsp. *Polymorphum* (*F. nucleatum*), *Porphyromonas gingivalis* (*P. gingivalis*), *Bifidobacterium animalis* subsp. *Lactis* Probio-M8 (Probio-M8), and *Clostridium perfringens* (*C. perfringens*) were anaerobically cultured for 12 h, and the rest were aerobically cultured for 12 h.

**Table 1:** List of indicator strains

**Indicator strain**	**Strain identification number**	**Cultivation conditions**
Gram-negative bacterium		
* Actinobacillus actinomycetemcomitans*	BNCC 336945	TSB, 37 °C
* Escherichia coli*	CICC 23657	LB, 37 °C
* Fusobacterium nucleatum* subsp*. polymorphum*	BNCC 336949	TSB, 37 °C
* Porphyromonas gingivalis*	BNCC 337441	TSB, 37 °C
* Pseudomonas aeruginosa*	ATCC 47085	LB, 37 °C
* Pseudomonas fluorescens*	CICC 21620	LB, 30 °C
* Riemerella anatipestifer*		LB, 37 °C
* Salmonella enterica*	CICC 10982	LB, 37 °C
* Vibrio harveyi*	CGMCC 18690	2216E, 37 °C
* Vibrio parahemolyticus*	CICC 21617	3% sodium chloride peptone water, 37 °C
Gram-positive bacterium		
* Bacillus cereus*	CICC 10277	LB, 37 °C
* Bifidobacterium animalis* subsp*. lactis*	Probio-M8	MRS, 37 °C
* Clostridium perfringens*	CICC 22949	Cooked meat medium base, 37 °C
* Lacticaseibacillus paracasei*	Zhang	MRS, 37 °C
* Lacticaseibacillus rhamnosus*	Probio-M9	MRS, 37 °C
* Lactiplantibacillus plantarum*	P-8	MRS, 37 °C
* Listeria monocytogenes*	ATCC 15313	LB, 37 °C
* Pediococcus acidilactici*	PA-19	MRS, 30 °C
* Staphylococcus aureus*	ATCC 12600	LB, 37 °C
* Streptococcus mutans*	BNCC 336931	BHI, 37 °C
Fungus		
* Aspergillus flavus*	CICC 2219	PDB, 30 °C
* Candida albicans*	CICC 32819	SDB, 30 °C
* Cladosporium sphaerospermum*	CICC 2477	PDB, 30 °C

*TSB* trypticase soy broth, *LB* LB broth, *2216E* 2216E liquid medium, *MRS* MRS broth, *BHI* brain heart infusion broth, *PDB* potato dextrose broth, *SDB* Sabouraud dextrose broth

### Antibacterial Activity Assay

Antimicrobial activity was assayed by the Oxford cup method using *Escherichia coli* (*E. coli*) CICC 23657 and *Staphylococcus aureus* (*S. aureus*) ATCC 12600 as the indicator strains [26]. Activated *L. paracasei* Zhang was inoculated in MRS medium (1%, V/V; pH 5.9), cultured for 24 h at 37 °C, and then centrifuged (4 °C, 6000 × *g*) for 10 min. Cell-free supernatants (CFS) were obtained and were filtered through a 0.22-µm pore-size membrane on an ultra-clean worktable. After determining the pH value of CFS, it was stored at 4 °C for use. The activated indicator bacteria were adjusted to 10^5^ CFU/mL with phosphate buffer saline, mixed with sterilized molten nutrient agar (1%, V/V), and poured (20 mL per assay) into fresh sterile plates. After the bacteria-inoculated agar solidified, Oxford cups were placed vertically on the surface of the culture medium and gently pressed to ensure good contact with the culture medium without gaps. For each assay, 150 µL of *L. paracasei* Zhang CFS was slowly added into the Oxford cups to allow pre-diffusion of the CFS at 4 °C for 4 to 10 h. The subsequent agar plates were incubated at 37 °C for 12 h for the development of inhibition zones. The diameter of the inhibition zone was measured by the linear measurement setting in a colony automatic counter. Each assay and measurement were performed in three replicates.

### Fractionation of CFS by Ultrafiltration

The *L. paracasei* Zhang CFS was first desalted by using electrodialysis equipment (GUOCHU TECHNOLOGY, Xiamen, China) until the electrical conductivity reached 3.0 ms/cm. Then, the desalted CFS was intercepted and subdivided by using ultrafiltration membranes of molecular weight cutoffs of 1, 3, 5, and 10 kDa, respectively (GUOCHU TECHNOLOGY, XiaMen, China). After that, the ultrafiltrates with different molecular weights were concentrated by spray drying (MOBILE MINOR, GEA Process engineering China Limited, ShangHai, China) with the following settings: initial material temperature, 20 °C; inlet temperature, 165 °C; exhaust temperature, 70 °C; inlet air volume, 8.0 m^3^/h; air pressure, 0.3 MPa; and feed speed, 8.0 r/min.

### Gel Filtration of 1–3 kDa CFS Fractions

The ultrafiltrate fractions of 1–3 kDa were further separated and purified by gel filtration chromatography using an AKTA Avant 150 (General Electric Company, Boston, USA). Briefly, the ultrafiltrate fractions of 1–3 kDa were diluted with ultrapure water to adjust to the concentration of 0.1 g/mL (m/v), and 6 mL of the diluent was loaded onto a Sephadex G25 column (2.6 × 100 cm; Pharmacia Biotech, Uppsala, Sweden). Then, the column was eluted with ultrapure water at a flow rate of 13 mL/min, and the eluent was monitored at an optical density of 280 nm (OD_280_). Fractions of 30 mL were collected according to the absorbance and 30-fold concentrated by lyophilization. Then, the high-absorbance fraction or the active fraction (AF) was assayed for antibacterial activities using the Oxford cup method against 23 indicator strains including Gram-positive and Gram-negative bacteria and fungus (Table 1).

### Tricine Sodium Dodecyl Sulfate Poly Acrylamide Gel Electrophoresis Analysis

The molecular mass of AF was determined by tricine sodium dodecyl sulfate poly acrylamide gel electrophoresis (Tricine-SDS-PAGE) according to the method of Schägger and Von Jagow with some modifications [27], and 4% stacking gel and 20% separating gel were prepared. The purified AF and a low molecular weight marker were run at 30 V for 100 min and 100 V during the rest of the separation. After electrophoresis, the gel was stained with Coomassie brilliant blue G-250 and destained with ethanol–acetic acid solution then imaged using the Gel imaging system (Bio-Rad, USA).

### Characterization of the Antibacterial Material in AF

#### Heat Stability

The heat stability of AF was tested. The AF was adjusted to pH 7.0 with 5 M NaOH. The osmotic pressure of the diluted AF was measured by an Automatic Cryoscopic Osmometer (OSMOMAT 3000, Gonotec GmbH, Germany) to exclude the effect of osmotic pressure on antibacterial activity. The AF was heated up for 30 min at different temperatures (40 °C, 50 °C, 60 °C, 70 °C, 80 °C, 90 °C, 100 °C, and 121 °C) before its antibacterial activities were determined by the Oxford cup method. Untreated AF was used as the control in the assay.

#### pH Stability

The acid–base stability of AF was tested. The AF was adjusted to pH 2, 3, 4, 5, 6, 7, 8, 9, and 10.0 using 3 mol/L HCL or 3 mol/L NaOH, respectively. The adjusted AF samples were incubated at 4 °C for 24 h before applying the Oxford cup method to assay changes in antibacterial activities. Subsequently, 0.9% sterile saline solution instead of AF was adjusted to the same pH and used as a control.

#### Enzyme Resistance

The resistance of the AF antibacterial material to various enzymes was tested, including catalase (pH 7.0, S10037, YuanYe Bio-Technology, Shanghai, China), α-amylase (pH 6.5, S10003, YuanYe Bio-Technology, Shanghai, China), lysozyme (pH 6.5, RL0295, Biocare, Zhuhai, China), RNAase (pH 7.6, R1030, Solarbio Science & Technology, Beijing, China), lipase (pH 8.0, S10035, YuanYe Bio-Technology, Shanghai, China), pronase E (pH 7.0, S10014, YuanYe Bio-Technology, Shanghai, China), pepsin (pH 2.0, RM1020, RYON, Shanghai, China), trypsin (pH 7.0, RM1022, RYON, Shanghai, China), papain (pH 7.0, RM1009, RYON, Shanghai, China), and protease K (pH 8.0, RM1060, YuanYe Bio-Technology, Shanghai, China). These enzymes were dissolved in ultrapure water at a concentration of 10 mg/mL, respectively. An aliquot of 100 µL of each enzyme was added to 900 µL of AF and incubated at 37 °C for 3 h. After that, the enzyme was inactivated by boiling for 10 min before conducting antibacterial assays by the Oxford cup method. The same volume of 0.9% sterile saline solution instead of AF was also treated with these enzymes and used as a control.

After the first round of assays, it was found that papain could inactivate the antibacterial activity of AF completely. Therefore, in order to further verify the effect of papain on the antibacterial activity of AF, the assay of AF resistance to papain was elaborated by incubating the AF-enzyme mix at 37 °C for 0, 0.5, 1, 3, and 6 h, respectively. After incubation for a specific time, the enzyme was inactivated by boiling for 10 min. Afterward, the antibacterial assay by Oxford cup method was performed.

Mining of *L. paracasei* Zhang Genome for Bacteriocin Gene Cluster.

The whole genome sequence of *L. paracasei* Zhang was retrieved from the NCBI database (accession number: CP001084.2) and searched through the BAGEL4 bacteriocin online database system (http://bagel4.molgenrug.nl) to identify potential bacteriocin gene cluster.

## Results

Antibacterial Activity of CFS of *L. paracasei* Zhang.

**Fig. 1:**
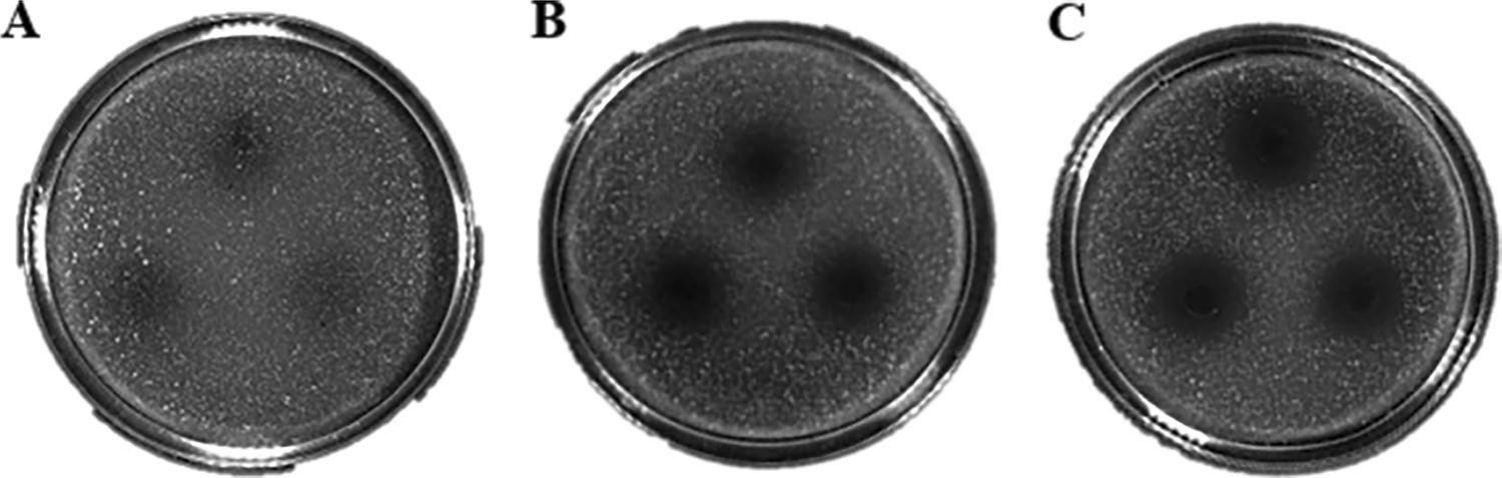
Antibacterial activity assays of *L. paracasei* Zhang metabolite. Cell-free supernatant on **A** *E. coli* and **B** *S. aureus*. **C** 1–3 kDa ultrafiltrate fraction on *S. aureus*

The CFS of *L. paracasei* Zhang showed a remarkable inhibitory effect against the indicator strain, *S. aureus* ATCC 12600, but not *E. coli* CICC 23657, indicating that *L. paracasei* Zhang could produce extracellular metabolites to inhibit certain bacteria (Fig. 1A and B). Ultrafiltration was then performed to enrich and desalt the active metabolites, obtaining ultrafiltrates of different molecular weights (i.e., fractions < 1 kDa, 1–3 kDa, 3–5 kDa, 5–10 kDa, and > 10 kDa, respectively). The different ultrafiltrate fractions were tested for their antibacterial activity against *S. aureus* ATCC 12600, and the 1–3 kDa fraction exhibited the strongest activity (Table 2). In addition, in the agar antibacterial activity assay, the inhibition zone diameter of 1–3 kDa fraction against *S. aureus* ATCC 12600 was obviously larger than that of the original CFS (Fig. 1C), indicating the enrichment of antibacterial active metabolites. The 1–3 kDa fraction was then further purified by gel filtration chromatography.

**Table 2:** Anti-staphylococcus activity of ultrafiltrates of cell-free supernatant of *L. paracasei* Zhang

**Molecular weight range (kDa)**	**Anti-staphylococcus activity**
< 1	−
1–3	++
3–5	+
5–10	+
> 10	−

“++” and “+” represent inhibition zone sizes of 11–15 mm and 7–11 mm, respectively; “−” means no inhibition

### Preliminary Purification of the 1–3 kDa Fraction

**Fig. 2:**
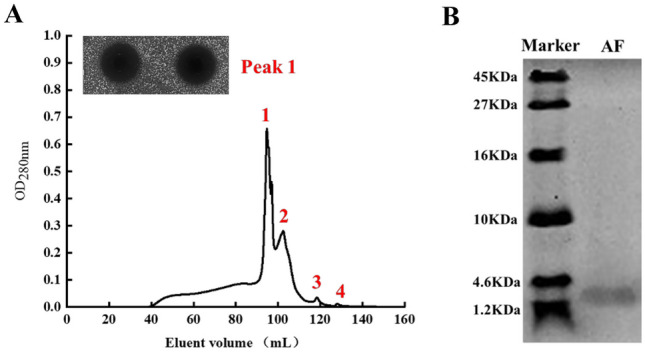
Preliminary purification and molecular mass estimation of AF. **A** Purification of AF by Sephadex G25 gel filtration chromatography. The inset shows the inhibition zones of peak 1 fraction against *S. aureus*. **B** The Tricine-SDS-PAGE image of AF of *L. paracasei* Zhang

The 1–3 kDa fraction was subjected to gel filtration chromatography. As shown in Fig. 2A, the extracellular metabolites of *L. paracasei* Zhang were eluted as four OD_280nm_ peaks. Fractions from the four peaks were collected and tested for their anti-staphylococcus activity, respectively, and only peak 1 was positive. This gel filtration fraction was regarded as the active fraction (AF) and was used for subsequent experiments. The purified AF analyzed by Tricine-SDS-PAGE showed a band between 4.6 KDa and 1.2 kDa (Fig. 2B).

### Characterization of the Antibacterial Activity of AF

#### Effect of Heat on the Antibacterial Activity of AF

**Fig. 3:**
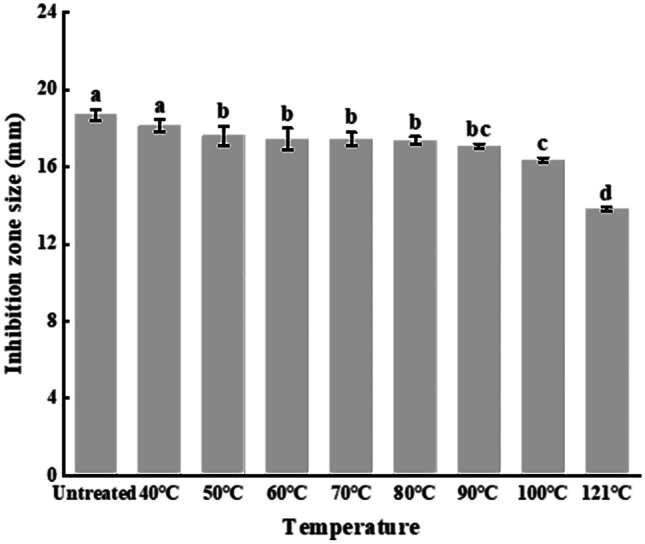
Thermal stability of the active fraction. Error bars represent standard deviation of the mean

Heat treatment is a common process in food production and other industrial processes, so it would be of interest to test the heat resistance of AF [28]. The inhibition zone diameter of heat-treated AF showed a decreasing trend, as the heat treatment temperature increased from 40 gradually to 100 °C. Even after harsh heat treatment for 30 min at 121 °C, the inhibition remained obvious with a zone size reduction of only 26.15% (Fig. 3), suggesting a high heat stability of the antibacterial metabolites.

As high osmotic pressure can inhibit bacterial growth, the osmotic pressure of tenfold-diluted AF (pH 7.0) was measured, which was about 800 mOsm/L, excluding the possibility of osmotic pressure interference on bacterial growth.

#### Effect of pH on the Antibacterial Activity of AF

**Fig. 4:**
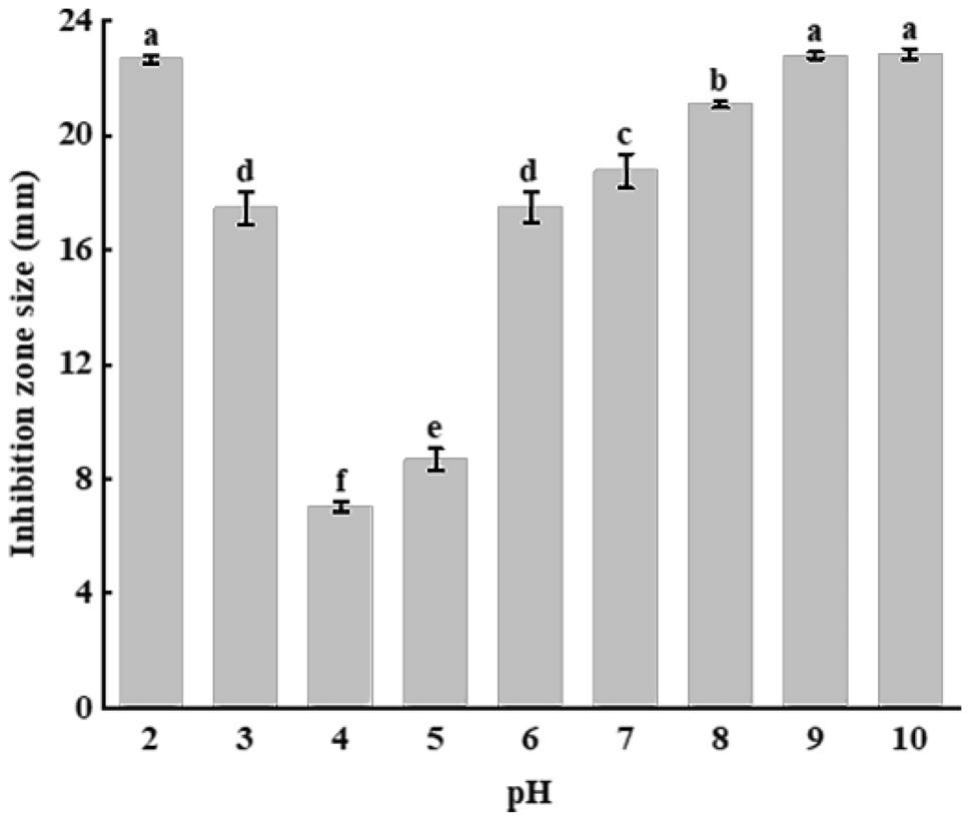
Antibacterial activity of the active faction at different pH ranges. Error bars represent standard deviation of the mean

Ideally, an industrial use biopreservative should have a good acid–base tolerance to ensure its stability in different matrix environments [29]. Thus, we tested the stability of the antibacterial activity of AF over a broad pH range (Fig. 4). Our results showed that it was greatly influenced by pH, with a drastic reduction in antibacterial activity against the staphylococcal indicator at pH 4 and 5 compared with a neutral pH (*p* < 0.0001). Interestingly, the antibacterial activity of AF was stronger in a highly acidic environment (pH 2) and in a moderate alkalinity range (pH 8–10) compared with a neutral pH (pH 7). These results showed that AF has a good acid–base tolerance.

#### Effects of Enzyme Digestion on the Antibacterial Activity of AF

**Fig. 5:**
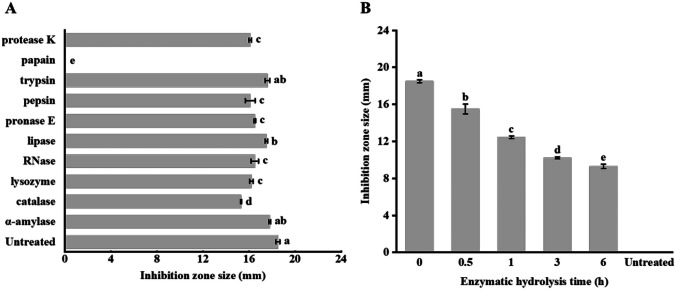
Effects of **A** different enzymes and **B** duration of papain treatment on the antibacterial activity of the active fraction. Error bars represent standard deviation of the mean

We then tested the stability of the antibacterial activity of AF in 10 different enzymes commonly found in food and human body environments. The results showed that papain was the only tested enzyme that could eradicate the antibacterial activity of AF, and the other nine applied enzymes had negligible effects on the antibacterial activity of AF (Fig. 5A), indicating that AF has strong enzyme tolerance. To further investigate the sensitivity of AF to papain, we performed a time-based bacteriostatic activity analysis of papain treatment (Fig. 5B). As expected, the antibacterial activity of AF decreased gradually with the increase of the time of enzymatic hydrolysis, indicating that the inactivation effect of papain was time-dependent. Based on these results, it is logical to speculate that the active antibacterial metabolite in AF is proteinaceous or peptide in nature.

#### Spectrum of Antibacterial Activity of AF

The AF exhibited varying degrees of antimicrobial activity when further tested against 20 indicator bacteria and three fungi (Table 3): strong inhibitory activity against *S. aureus* (ATCC 12600); moderate inhibitory activity against *P. fluorescens* CICC 21620 and *P. aeruginosa* ATCC 47085; weak inhibitory against *A. actinomycetemcomitans* BNCC 336945, *L. monocytogenes* ATCC 15313, and *B. cereus CICC 10277*; and no inhibitory activity against the three tested fungi and some common LAB and/or probiotic bacteria such as *L. plantarum* P-8, *L. rhamnosus* Probio-M9, *P. acidilactici* PA-19, *B. animalis* subsp*. lactis* Probio-M8, and *L. paracasei* Zhang.

**Table 3:** Antibacterial activity spectrum of *L. paracasei* Zhang active fraction

**Indicator strain**	**Strain identification number**	**Inhibition activity**
Gram-negative bacterium		
* A. actinomycetemcomitans*	BNCC 336945	+
* E. coli*	CICC 23657	−
* F. nucleatum* subsp*. polymorphum*	BNCC 336949	−
* P. gingivalis*	BNCC 337441	−
* P. aeruginosa*	ATCC 47085	++
* P. fluorescens*	CICC 21620	++
* R. anatipestifer*		−
* S. enterica*	CICC 10982	−
* V. harveyi*	CGMCC 1.8690	−
* Vibrio parahemolyticus*	CICC 21617	−
Gram-positive bacterium		
* B. cereus*	CICC 10277	+
* B. animalis* subsp*. lactis*	Probio-M8	−
* C. perfringens*	CICC 22949	−
* L. paracasei*	Zhang	−
* L. rhamnosus*	Probio-M9	−
* L. plantarum*	P-8	−
* L. monocytogenes*	ATCC 15313	+
* P. acidilactici*	PA-19	−
* S. aureus*	ATCC 12600	+++
* S. mutans*	BNCC 336931	−
Fungus		
* A. flavus*	CICC 2219	−
* C. albicans*	CICC 32819	−
* C. sphaerospermum*	CICC 2477	−

“+++”, “++”, and “+” represent inhibition zones > 15 mm, 11–15 mm, and 7–11 mm, respectively; “−” means no inhibition

Mining for *L. paracasei* Zhang Bacteriocin Gene Cluster.

**Fig. 6:**
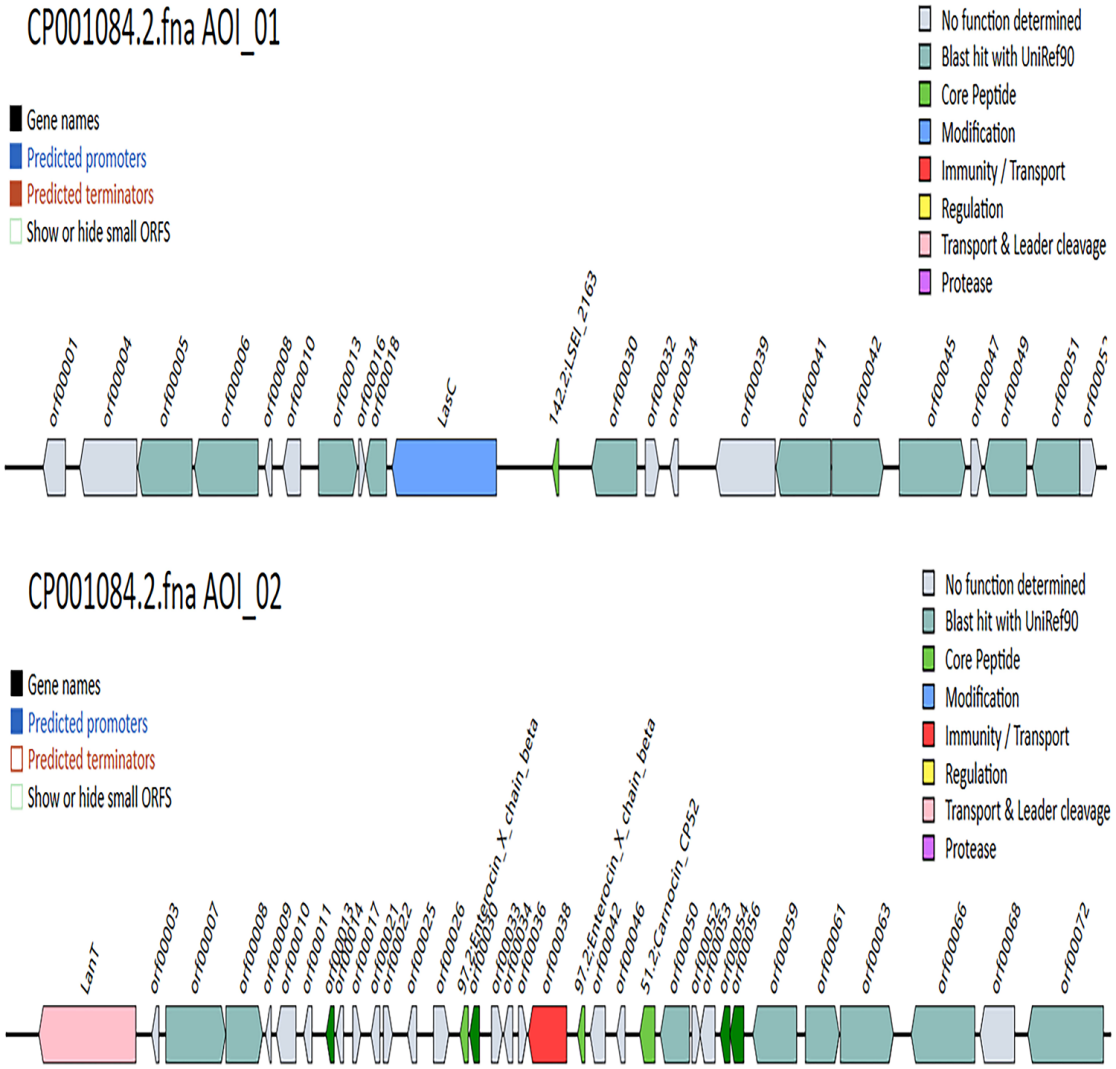
Schematic diagrams showing the two genomic areas of interest (AOI) in *Lacticaseibacillus* (*L.*) *paracasei* Zhang, identified by BAGEL4. These regions were predicted to possess putative bacteriocin gene clusters. The AOI_1 and AOI_2 comprise 52 and 72 open reading frames, respectively, but small open reading frames are hidden

BAGEL4 is an online platform that facilitates data mining of bacterial (meta-)genomic DNA for bacteriocins, as well as other bacterial ribosomally synthesized and post-translationally modified peptides [30]. The BAGEL4 search predicted two areas of interest (AOI), where putative gene clusters for antibacterial substances biosynthesis are located in the *L. paracasei* Zhang genome (Fig. 6). Noteworthy, these potential bacteriocins are possibly involved in the antagonistic activities reported in this work.

AOI_1 (chromosome position from 2079925 to 2100039) contained 52 open reading frames (ORFs) (Table 4). One core gene was predicted to share 50.98% homology with LESI_2163, and a lantibiotic modifying enzyme-encoding gene (*LasC*) harboring a PF00733 domain was predicted. BLASTP analysis indicated that this gene encodes a protease that catalyzes the conversion of aspartate to asparagine. Other predicted gene features in AOI_1 included transcription regulatory factors, transposase, and phosphotransferase system-related genes.

**Table 4:** Functional annotation of gene clusters predicted by BAGEL4

**No.**	**Functional**	**Accession (ID)**
AOI_1		
ORF00005	D-lactate dehydrogenase	sp|Q88VJ2|LDHD_LACPL
ORF00006	Probable N-acetyl-LL-diaminopimelate aminotransferase	sp|P16524|DAPX_BACSU
ORF00013	Uncharacterized protein M6_Spy0233	sp|Q5XDZ5|Y233_STRP6
ORF00018	Uncharacterized HIT-like protein MG132	sp|P47378|YHIT_MYCGE
LasC	Asparagine synthetase [glutamine-hydrolyzing] 1	sp|P54420|ASNB_BACSU
142.2;LESI_2163		
ORF00030	Phosphatidylglycerol lysyltransferase	sp|C0H3X7|MPRF_BACSU
ORF00041	1,4-dihydroxy-2-naphthoate octaprenyltransferase	sp|P39582|MENA_BACSU
ORF00042	Hexaprenyl-diphosphate synthase large subunit ((2E,6E)-farnesyl-diphosphate specific)	sp|O66129|HEXB_MICLU
ORF00045	Acetate kinase	sp|Q03CP2|ACKA_LACP3
ORF00049	Uncharacterized transposase-like protein HI_1721	sp|O05086|Y1721_HAEIN
ORF00051	Insertion element IS1223 uncharacterized 20.7 kDa protein	sp|Q48585|YI3A_LACJH
AOI_2		
LanT	ATP-binding protein ComA	sp|P59653|COMA_STRR6
ORF00007	Bacteriocin production-related histidine kinase	Q1U9H1_LACRE
ORF00008	Response regulator PlnD	CAA64207.1
ORF00013	ggmotif; lactococcin	
97.2;enterocin_X_chain_beta	ComC; lactococcin; bacteriocin_IIc	
ORF00030	ComC; bacteriocin_IIc	
ORF00038	P71468_LACPL PlnI (immunity protein PlnI, membrane-bound protease CAAX family)	P71468
97.2;enterocin_X_chain_beta	Bacteriocin_IIc	
51.2;carnocin_CP52	Carnocin	
ORF00050	Probable maltose O-acetyltransferase	sp|P37515|MAA_BACSU
ORF00054	Bacteriocin_IIc	
ORF00056	Bacteriocin_IIc	
ORF00059	Oxidoreductase YdhF	sp|P76187|YDHF_ECOLI
ORF00061	Uncharacterized oxidoreductase SSP1627	sp|Q49WS9|Y1627_STAS1
ORF00063	Probable NADH-dependent flavin oxidoreductase YqiG	sp|P54524|YQIG_BACSU
ORF00066	Fumarate hydratase class II	sp|Q929E8|FUMC_LISIN
ORF00072	Divalent metal cation transporter MntH	sp|Q38UX8|MNTH_LACSS

AOI_2 (from 2311300 to 2335548) contained 72 ORFs with two potential bacteriocin genes as the core genes (Table 4). The two genes were predicted to share 64.00 and 31.82% homology with two bacteriocin-encoding genes, namely enterocin_X_chain_beta and carnocin_CP52, respectively. Besides, within AOI_2, several other ORFs (OR00030, ORF00054, and ORF00056) showed homology to some known class II bacteriocin genes with double-glycine leader peptide, and ORF00030 was homologous with ComC bacteriocin. Other bacteriocin-related gene features were also identified within the AOI_2 region, including ORF00013, encoding a putative bacteriocin immunity protein, characterized by its specific spatial conformation and functional structural motifs; ORF00007 and ORF00008, encoding a histidine kinase associated with bacteriocin production and a *PlnD*-liked response regulator for negative regulation of bacteriocin synthesis, respectively; and ORF00038, encoding a cognate immunity protein-containing CAAX-like protease, which was homologous to *PlnI* (P71468_LACPL). Moreover, the region of AOI_2 also encoded a putative ABC transporter gene and a helper factor gene that were homologous to *LanT* and *HlyD*, respectively.

## Discussion

During the growth and metabolism of LAB, a variety of bioactive metabolites, such as short-chain fatty acids, organic acids, diacetyl, peptides, hydrogen peroxide, extracellular polysaccharides, and bacteriocins, are produced and extracellularly secreted. They play important roles in cellular signaling, and they are the bioactive metabolites that confer probiotic effects on the host [31]. This study preliminarily purified and characterized the antibacterial AF in the CFS of *L. paracasei* Zhang. We also mined the genome of *L. paracasei* Zhang for genomic regions–encoding putative bacteriocins.

Normally, the concentrations of some active metabolites are too low to be detected by traditional in vitro assays [32, 33]. Therefore, it is necessary to enrich the bioactive fraction for further experiments. Herein, a two-step purification was implemented by ultrafiltration and Sephadex G-25 gel filtration chromatography, which mainly desalted the CFS and concentrated the active metabolites based on size. By testing the antibacterial activity of the ultrafiltrates, we found that the bioactivity was concentrated in the 1–3 kDa fraction, which was further purified by gel filtration chromatography and subsequent analysis. In the purification process, small-size metabolites like lactic acid and acetic acid would be separated from the active metabolites, as most of these interfering molecules, ions, and acid salts had a molecular size of smaller than 200 Da. Moreover, we also ensure the osmotic pressure of the diluted AF was in a range not interfering with bacterial growth in the agar antimicrobial assays.

Our further assay on the antimicrobial spectrum of AF revealed a broad antibacterial spectrum, inhibiting both members of the Gram-positive (*S. aureus*, *L. monocytogenes*, *B. cereus*) and Gram-negative (*P. fluorescens*, *P. aeruginosa*, and *A. actinomycetemcomitans*) bacteria. The AF exhibited the strongest inhibitory activity against *S. aureus* compared with other tested bacteria. *S. aureus* is both a food and human pathogen. As a foodborne pathogen, *S. aureus* is widely distributed in air, water, and different kinds of food and can cause food poisoning by secreting enterotoxin. On the other hand, *S. aureus* is also widely distributed on the skin surface, larynx, nasal cavity, and other mucosal surfaces; it does not only cause skin infection but also induces infective endocarditis, fasciitis, osteomyelitis, and pneumonia occasionally, posing a great threat to human health and safety [33]. Thus, the strong inhibitory activity of AF against *S. aureus* is of interest for it to be applied in food preservation and products like cosmetics. Another feature of AF is that it exerted no inhibitory effect on LAB or probiotics, which is similar to other LAB-originated bacteriocins [34, 35]. This makes AF a desirable food biopreservative, which would not alter the endogenous and beneficial gut microbiota when applied to food.

Apart from a wide antibacterial spectrum, a suitable bacteriocin should ideally have good tolerance to heat, acid–base fluctuation, and common enzymes like proteases [36]. We found that the antibacterial activity of AF has a high stability over a wide range of temperatures (40 to 100 °C) and pH (pH 2–3 and pH 6–10). The good thermal and acid–base stability makes it a suitable biopreservative for harsh and complex industrial production processes. Additionally, the bioactivity of AF was highly resistant to digestion by a variety of common industrially used enzymes and enzymes present in animal/human body (such as trypsin, pepsin, α-amylase, and protease K), except for papain, which differs from the high susceptibility of many previously characterized bacteriocins and most antibacterial peptides [18, 34, 35, 37, 38]. Trypsin and pepsin are two of the most important digestive proteases existing in the human gastrointestinal tract, and protease K and α-amylase are often used in the food industry. The time-based kinetic analysis of papain digestion of the AF in the current study revealed that papain inactivation was a gradual process, suggesting that the nature of the antibacterial metabolite is protein or peptide. In conclusion, the good stability and strong resistance to different kinds of enzymes make AF suitable for use in food preservation and as an oral supplement against gastrointestinal pathogens [39].

In this study, several putative bacteriocin-like genes were identified in *L. paracasei* genome with the BAGEL 4 web server, including LESI_2163 in AOI_1 and enterocin_X_chain_beta ( *E*-value = 1.88 e^−09^, match = 64.000%), carnocin_CP52 ( *E*-value = 1.39 e^−20^, match = 31.818%), ORF13, ORF30, ORF54, and ORF56 in AOI_2. Previous studies have shown that the mature peptide of LSEI_2163 was a class IId bacteriocin that exhibited antimicrobial activity against some lactobacilli and several *Listeria* species [40]. The sequence of LSEI_2163 was found to be 100% identical to those sequences present in several strains of *L. paracasei*, including ATCC 334 (accession number, CP000423.1), TD 062 (accession number, CP044361.1), and TCS (accession number, CP038153.1). Enterocin X_chain_beta, a class IIc bacteriocin, belongs to the *Lactococcus* protein-like family and has 50.98% homology with Enterocin X from *Enterococcus faecium* KU-B5. Enterocin X is a heat-resistant dipeptide bacteriocin that contains non-thiopeptides, and its full antibacterial activity requires the interaction of two complementary peptides [41]. BLASTN analysis showed that the amino acid sequence was 100% identical to that of *L. paracasei* (CP032637.1). Carnocin_CP52 is homologous to carnobacteriocin B2, which is the first bacteriocin identified from a strain of *C. piscicola* isolated from a dairy product and identified as class II bacteriocin [42]. Blast results by UniRef90 revealed that ORF13 belongs to the lactococcin-like family and contains ggmotif, and ORF30, ORF54, and ORF56 are class II bacteriocins containing double-glycine leader peptides. As is known to all, cell density–dependent gene expression in bacteria exists widely and is mediated by extracellular communication molecules. Previous studies have found that gram-positive bacteria usually perceive population density through post-translational processing of peptide pheromones [43]. The Class II bacteriocins are usually produced as propeptides, which contain a characteristic amino-terminal leader sequence called a double-glycine leader sequence [44]. Three components are involved in the regulation of class II bacteriocin production—an inducible peptide, also known as a pheromone, and a two-component regulatory system [45]. Similar to the bacteriocin peptides, the inducing peptide is cationic, synthesized as a propeptide with a double-glycine leader sequence, which is cleaved during transportation. The two-component system includes a membrane-bound histidine protein kinase that acts as an environmental sensor and a cytoplasmic response regulator that is a DNA-binding protein responsible for the activation of the transcription of its target genes. After secretion, the inducing peptide is subsequently sensed by the dedicated two-component system, thereby inducing the transcription of the operons involved in bacteriocin production [43]. As we know, functional bacterial gene clusters for producing extracellular cationic peptide-bacteriocins are usually organized as operons that comprise a complete set of genes for production until externalization of the bacteriocin, including at least four gene components: bacteriocin structural gene, specific immune protein gene, ABC transporter gene, and its accessory protein gene [46]. Therefore, based on the predicted gene function, it is likely that AOI_02 but AOI_01 was functional in biosynthesizing bacteriocins that are responsible for the seen antibacterial activity of *L. paracasei* Zhang subjected to the growth environment and quorum sensing activity. What is more, amino acid sequence alignment results showed that all of ORF13, ORF30, ORF54, and ORF56 had lower similarity with the reported bacteriocins of other strains of *L. paracasei*, including *L. paracasei* ZFM54 [18], *L. paracasei* LS-6 [47], and *L. paracasei* HD1-7 [48], indicating the most possibility of production of novel bacteriocins by *L. paracasei* Zhang.

Apart from the conventional use of bacteriocin as a food preservative, it has been proposed to expand the applications of bacteriocins from food to health. For example, nisin has shown a cytotoxic effect on the SW480 cancer cell line, inducing apoptosis by increasing the ratio of bax/bcl-2 on both mRNA and protein levels [49]. Bacteriocins have also been proposed as potential anti-cancer agents due to their selective action against cancer cells based on distinctive cell membrane differences between healthy and cancer cells. Moreover, antibacterial metabolites, including extracellular polysaccharides, bacteriocins, and antimicrobial peptides, have been found to exert beneficially regulating gut microbiota composition, improving host immune response, and enhancing intestinal barrier function. For example, a study found that adding AMP Gal-13 to the diet of broilers could improve the intestinal digestive capacity, antioxidant activity, and immune function of broilers, ultimately promoting the growth of broilers [50]. These active metabolites can stimulate tissue development and affect the nutritional level and physiological function of the body. Another study found that adding nisin to the diet of broilers could reduce potential jejunal and cecal pathogens, such as *Clostridium perfringens* and *Enterobacteriaceae*, and substantially suppress jejunal bacterial fermentation [51]. The addition of nisin and Chinese gallnut to the diet of carp substantially remodulated the intestinal microbiota, suppressing amoeba and enhancing *Bacteroides* [52].

The *L. paracasei* Zhang strain has shown multiple probiotic functions in animal and human intervention trials, including the enhancement of antioxidation and anti-lipid peroxidation effects [21], stimulating cellular and humoral immunity and tumor immunity [22], improving blood lipids and liver lipid metabolism [23], preventing type II diabetes, protecting liver and preventing liver injury [24], and regulating the gut microbiota via increasing the beneficial microbes while reducing potential pathogens [21]. In this study, we only confirmed that the CFS of *L. paracasei* Zhang contained antibacterial metabolites, probably class II bacteriocins, and speculated based on genomics prediction the molecular size and physico-chemical properties (acid–base, thermos-, and enzyme tolerance) of the bioactivity. Whether these metabolite(s) and/or bacteriocin serve other biological functions in vitro and in vivo remains to be further explored. Future studies should also focus on large-scale purification and identification of the bioactive metabolites, exploring their effectiveness in food preservation and suppression of gastrointestinal infection by pathogens, and elucidating the mechanism of the bioactivity.

From these characteristics, it can be concluded that the active substance in AF is a novel antibacterial metabolite. It is believable that the two outstanding advantages will promote and broaden its application in food preservation and pathogen infection.

## Conclusions

This study preliminarily purified and confirmed that the 1 to 3 kDa fraction of CFS of *L. paracasei* Zhang exhibited a broad spectrum of antibacterial activity against multiple foodborne and human pathogens, and food spoilage bacteria. Based on the genomics prediction and the physico-chemical nature of the antibacterial activity, the bioactive metabolite is possibly a class II bacteriocin. The bioactivity has good tolerance to heat, acid–base change, and digestion by various enzymes, making it a potential candidate for use in food preservation and an agent for suppressing gastrointestinal infections.

## Data Availability

No datasets were generated or analysed during the current study.
